# A novel alveoli-on-chip platform for modeling cyclic stretch in patient-derived alveolar epithelial cells cultured from organoids

**DOI:** 10.1039/d5lc00473j

**Published:** 2025-10-24

**Authors:** Mohammad Amin Hajari, Jan Schulte, Dario Principi, Damian Schnidrig, Sabine Schneider, Tobias Weber, Joo-Hyeon Lee, Patrick Dorn, Pauline Zamprogno, Thomas Michael Marti, Olivier T. Guenat

**Affiliations:** a Organs-on-Chip Technologies Lab, ARTORG Center, University of Bern Bern Switzerland olivier.guenat@unibe.ch; b Graduate School for Cellular and Biomedical Sciences (GCB), University of Bern Bern Switzerland; c Cambridge Stem Cell Institute, Jeffrey Cheah Biomedical Centre, University of Cambridge Cambridge CB2 0AW UK; d Developmental Biology Program, Sloan Kettering Institute, Memorial Sloan Kettering Cancer Center New York NY 10065 USA; e Department of BioMedical Research, University of Bern Bern Switzerland; f Department of General Thoracic Surgery, Inselspital, University Hospital of Bern Bern Switzerland; g Department of Pulmonary Medicine, Inselspital, University Hospital of Bern Bern Switzerland

## Abstract

We report a novel alveoli-on-chip (AOC) platform that replicates an array of alveoli closely matching *in vivo* dimensions and enables three-dimensional cyclic stretching to simulate the respiratory movements experienced by the alveolar epithelium. This system is based on an arrangement of parallel pneumatic channels with a sinusoidal wall morphology leading to interconnected circular chambers with a 250 μm radius. Above the pneumatic channels is a thin flexible membrane on which alveolar epithelial cells are cultured. When pressure is applied, the membrane deforms, with maximum deformation in the centre of these chambers. To simplify cell culture management, the AOC has an open design and is configured for medium throughput in a 24-well plate format. The chip is populated with patient-derived lung alveolar epithelial cells from organoids. Type 2 lung alveolar epithelial cells (AT2) were magnetically sorted from tissue samples obtained from patients undergoing lung surgery and cultured as organoids for expansion. The expansion efficiency of AT2 patient-derived organoids was optimized by the supplementation of endothelial supplied alveolar potentiators (ESAP), resulting in a 2.5-fold increase in organoid size increase after 10 days of culture compared to without ESAP. Immunostaining of expanded organoids confirmed their epithelial identity (EpCAM^+^) and demonstrated the presence of AT2s (SFTPC^+^, HTII-280^+^), AT1s (PDPN^+^), and transitional AT0s (KRT8^+^) subtypes. Dissociated lung organoids cells from three patients were cultured on the chip under conditions with or without physiological mechanical stress. Bulk RNA sequencing revealed that cyclic stretch significantly influences gene expression in lung alveolar epithelial cells. An increase in the expression of genes associated with autophagy, G2/M checkpoint and mTORC1 was observed, indicating cellular stress responses and adaptation to mechanical stimuli. Thus, this study not only showcases the AOC's potential to recapitulate alveolar epithelium mechanical cues but also offers a robust and scalable platform to be used for research and preclinical applications.

## Abstract 2

This alveoli-on-chip (AOC) mimics breathing motions in a 24-well format and supports patient-derived lung organoids with enhanced growth. Cyclic stretch alters gene expression, offering a scalable tool for lung biology and preclinical studies.

## Graphical abstract

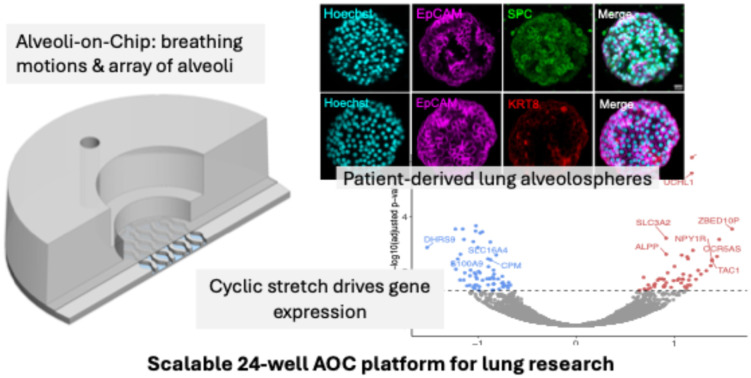

## 1. Introduction

The success of lung-on-chip systems lies in their ability to replicate key aspects of the alveolar environment, particularly the air-blood barrier and the dynamic mechanical forces associated with breathing motions.^1,2^ These platforms have provided valuable insights into how mechanical cues influence pulmonary physiology and alveolar epithelial cell behavior. Studies have for instance demonstrated that cyclic mechanical stretch can promote alveolar epithelial cell differentiation,^3^ activate the ERK signaling pathway,^4^ increase the alveolar barrier's sensitivity to toxic agents,^5^ impair wound healing,^6^ and enhance surfactant protein expression^7^—underscoring the pivotal role of mechanical forces in lung function.

Despite these advances, early lung-on-chip models oversimplified alveolar biomechanics by neglecting essential structural and functional features such as alveolar size, curvature, and the complex deformation patterns associated with breathing.^8^ Breathing was often represented as a uniform, linear stretch of the alveolar epithelium,^2^ which does not reflect the intricate micromechanical behavior of the lung. More recent developments have sought to overcome these limitations by introducing alveolar arrays that better replicate the geometry^9,10^ and curvature^11^ of native alveoli. Additionally, PDMS membranes commonly used in early lung-on-chips^1,2^ have been replaced with protein-based, biodegradable membranes,^9,10^ offering stiffnesses that more closely approximate those found *in vivo*.^12^ These improvements bring lung-on-chip models closer to physiological relevance.

Crucially, *in situ* observations and computational simulations have revealed that alveolar deformation during respiration involves a sequence of mechanical events—beginning with alveolar unfolding and followed by the development of spatially heterogeneous stress gradients across the alveolar surface.^8,13^ These findings demonstrate that the true mechanical environment of the alveolar epithelium is far more complex than simple uniaxial stretching.

Another unresolved challenge in lung-on-chip development is the choice of alveolar epithelial cell source. Adenocarcinoma-derived cell lines, such as A549, are widely used but fail to accurately represent the phenotype and function of healthy alveolar epithelial cells. These cell lines often exhibit substantial heterogeneity and phenotypic plasticity,^14^ limiting their relevance for modeling normal lung biology. In contrast, primary alveolar epithelial type II cells (AT2) more closely resemble native lung cells but are difficult to expand due to their low proliferative capacity and limited availability. Lung organoids derived from patient tissues offer a promising alternative,^15^ and can be dissociated for culture on-chip.^16^ Yet they are also constrained by the slow proliferation of AT2—often requiring several weeks to generate sufficient quantities for experimentation. This significantly hinders the development of physiologically relevant lung-on-chip models.

In addition, the throughput of current lung-on-chip platforms—both commercial and custom-built—remains limited, typically ranging from just one well^2^ to 12 wells^17^ per device, which restricts scalability and limits the ability to perform parallel experiments.

We present a novel alveoli-on-chip (AOC) platform featuring an array of alveoli that closely match *in vivo* dimensions and undergo cyclic stretching in three directions to replicate physiological mechanical stress gradients. The platform is user-friendly, with an open-top design embedded in a standard 24-well plate, ensuring compatibility with conventional cell culture workflows. To overcome the limited availability of primary human alveolar epithelial cells, we optimized an organoid culture protocol using patient-derived cells, cutting the time to generate alveolospheres by half. We then performed immunostaining to characterize the cellular composition of the expanded organoids and similarly analyzed the composition after dissociation and seeding onto the AOC device. Finally, we performed bulk RNA sequencing on AT2 organoid-derived cells from three patient samples to evaluate their transcriptional response to physiologically relevant cyclic mechanical stress.

## 2. Methods and materials

### 2.1 Fabrication of the novel alveoli-on-chip

The design of the novel alveoli-on-chip was done using Solidworks 2022. From this, a PDMS master mould was fabricated as a wafer using SU-8. This master mould was subsequently replicated with epoxy resin (WEICON C, Plastic Metal) for enhanced durability and to increased production. AOCs were manufactured *via* PDMS casting (SYLGARD 184, Silicone Elastomer Kit) at a polymer-to-curing agent ratio of 10 : 1 in between two epoxy resin molds, in one piece forming the pneumatic and culturing compartment, as well as the culturing membrane. The casting time at 60 °C was consistently kept at 18 h. The assembly of the AOCs into a multiwell plate (12 or 24 Well glass bottom plate, Cellvis), led to the creation of the final AOC-plate in a 12 or 24 well configuration, each plasma bonded onto the well-plates glass bottom. A 30-minute post-bake period was employed at 60 °C to reinforce bonding.

### 2.2 Mechanical characterisation of the novel AOC

The mechanical behaviour of the AOC's membrane deflection was characterised through a series of pressure-deflection tests. These tests were conducted on AOCs that were fabricated and assembled according to previously established protocols. Once fully assembled, the chips were connected to an OB1 microfluidic flow controller (Elveflow) using 2 mm plastic tubes (Festo). The membrane deflection of the AOCs was measured under a Zeiss AxioImager microscope. This involved assessing *z*-plane displacement across a pressure range from 0 to 800 mbar, with incremental steps of 50 mbar. To simulate physiological conditions, measurements were also conducted at 37 °C in a pre-warmed incubation chamber integrated surrounding the microscope. Furthermore, to evaluate the impact of prolonged incubation on membrane deflection, the AOCs underwent a pre-culturing phase at 37 °C for 5 days inside an incubator. Subsequent measurements were taken every two days over a 10-day period. During this time, the AOCs were subjected to cyclic pressure at 400 mbar with a frequency of 0.25 Hz. This methodology allowed for a comprehensive understanding of the AOCs' mechanical properties under various conditions within a realistic biological experimentation setting.

### 2.3 Numerical simulation of the pneumatic membrane deformation

The numerical simulation of the AOC mechanical properties was done using Finite Element Analysis (FEA) in Abaqus CAE software under an academic research license from the University of Bern. The analysis utilised a local model of one alveolus designed to align closely with the experimental data, focusing on the pillar and membrane pattern of the chip. The local model comprised a 3D solid representing one-quarter of an alveolus, exploiting its symmetrical structure for an increased mesh density. A 2D sensing layer on the membrane was integrated to analyse the in-plane strain experienced at the culturing surface. An isotropic neo-Hookean hyperelastic material model (*C*_0_ = 2.8 MPa, *D* = 0 MPa)^55^ was used for the 3D part to accommodate larger strains, and an isotropic linear elastic model for the 2D layer (Young's modulus of 0.161 MPa, Poisson's ratio of 0.4999). Both included long-term viscoelasticity settings and accounted for geometric non-linearity, allowing for the analysis of large deformations. Boundary conditions involved embedding the base of the pillar and applying symmetry axes. The load was progressively increased from 0 to 800 mbar over 8 seconds, applied under the membrane and against the edge of the pillars. A mesh convergence analysis was conducted for the alveolus to ensure accuracy in the results for deflection magnitude and maximum principal strain. The mesh sizes were set to 0.0125 mm for both the 3D structure and 2D layer, with C3D10H elements for 3D and triangular elements for the 2D layer.

### 2.4 Clinical sample, patient consent and ethical agreement

The lung tumour samples were obtained from patients after surgical resection of non-small cell lung cancer (NSCLC) at University Hospital of Bern (Thoracic Surgery Department). All patients gave their written consent to use the surgical material for research purposes, which was approved by the Ethics Committee of the Canton of Bern (KEK-BE:2018-01801). All procedures were performed in accordance with the institutional guidelines of the Canton of Bern. In total, biopsies from seven donors were used to generate and expand alveolospheres, with cells from three patients cultured on the AOC chip.

### 2.5 Isolation of patient-derived AT2

The isolation and culture protocol was adapted from Youk *et al.* 2020^21^ (media formulation) and Zacharias and Morrisey 2018^56^ (MACS protocol). AT2 were isolated from histologically normal lung tissue adjacent to resected lung cancers, confirmed to be uninvolved by the tumor. Before the dissociation of resected human lung tissue, it was washed or stored overnight in PBS containing 1% v/v P/S. The tissue was minced into 1 mm pieces using scissors and washed once with PBS. Followed by enzymatic dissociation for 1 h at 37 °C during constant agitation in pre-warmed dissociation buffer containing Dispase II (2U mL^−1^, Sigma-Aldrich), Collagenase 4 (1 mg mL^−1^, Worthington) and 0.1 mg/mL DNase I (Sigma-Aldrich) in PBS. The suspension was pre-filtered through a sterile cloth with coarse fabric, filtered through a 100 μm cell strainer and washed with 10 ml of cold MACS buffer containing 1% v/v FBS and 1% v/v P/S in PBS. Cells were centrifuged at 450 rcf and 4 °C for 10 min, followed by careful aspiration. Resuspending in 10 ml RBC lysis buffer (BioLegend), the pellet was incubated for 10 min at RT. To quench the reaction, the cells were washed 3× with MACS buffer at 450 rcf and 4 °C for 5 min. Cells were counted using a Luna II cell counter and then incubated in 2 ml HTII-280 mouse IgM antibody (1 : 50, Terrace Biotech) for 30 min at 4 °C. After two washes with MACS buffer at 450 rcf and 4 °C for 5 min, the pellet was resuspended with anti-mouse IgM MicroBeads (1 : 5, Miltenyi Biotec) in PBS, for 30 min at 4 °C. The magnetically labelled cells were washed again, resuspended in 2.5 ml MACS buffer and transferred into a 5 ml polystyrene tube. This tube was placed inside an EasySep™ magnet (Stemcell Technologies) and let to rest for 5 min at RT. In a continuous motion, the tube and magnet were inverted and left inverted for 2–3 seconds to isolate labelled cells and let unlabelled cells flow trough. The isolated cells were set aside and the flow through solution underwent the same isolation procedure again to retrieve remaining labelled cells. The two isolation solutions were combined and constituted the HTII-280 enriched population. To increase the yield, the enrichment was followed by a final sorting. Following dissociation and sorting, each isolation typically yielded ∼1.5–2.5 million cells. The solution containing sorted cells could then be used for subsequent seeding or analysis.

### 2.6 FACS of AT2 cells

Freshly dissociated, flow-through, enriched and final sorted cells, labelled for HTII-280, were incubated for 30 min with an anti-mouse IgM AF488 antibody. Afterwards they were washed 3× with MACS buffer at 450 rcf and 4 °C for 5 min. The labelled cell solutions were then analysed using a BD SORP LSR II cytometer. The cell sorting results were used to determine the number of HTII-280 positive cells within the respective solutions.

### 2.7 Culture of AT2 organoids

HTII-280^+^ cells were isolated from surgical resected human lung tissue and resuspended in base medium (Advanced DMEM/F12 (Thermo Fisher Scientific) supplemented with 10 mM HEPES (Sigma-Aldrich), 1 μg mL^−1^ Primocin (Invivogen), 1 mM N-Acetylcysteine (Sigma-Aldrich), and 10 mM Nicotinamide (Sigma-Aldrich)) at a concentration of 1 × 10^6^ cells ml^−1^. The cell suspension was mixed 1 : 1 with Growth-Factor-Reduced (GFR) Matrigel (CORNING) on ice and added as 50 μl domes (50 000 cells per dome) into a 24-well plate. After 30 min of solidification at 37 °C, 500 μl of warmed alveolar medium (base medium supplemented with 1 × B27 (Thermo Fisher Scientific), 50 ng ml^−1^ recombinant human EGF (PEPROTECH), 100 ng ml^−1^ human FGF7/KGF (PEPROTECH), 100 ng ml^−1^ human FGF10 (PEPROTECH), 100 ng ml^−1^ NOGGIN (PEPROTECH), 10 μM SB431542 (Sigma-Aldrich) and 3 μM CHIR99021 (Sigma-Aldrich) were added to each well. During the first 48 h, alveolar medium was supplemented with Y-27632 (10 μM, Sigma-Aldrich), after which the cells were maintained at 37 °C and 5% CO_2_. The medium was changed every 2–3 days. Organoid formation and maturation took place over 3–5 weeks, depending on the source material, and were passaged by adding 500 μl of 2U mL^−1^ Dispase II (Sigma-Aldrich) for 45 min at 37 °C, followed by washing in MACS buffer at 350 rcf for 5 min. The released organoids were dissociated with TrypLE (Thermo Fischer) for 10 min, washed, and collected. Each Matrigel dome produced on average ∼250 000–350 000 epithelial cells comprising AT2, AT1, and AT0 populations, which were subsequently either cryopreserved, further expanded, or seeded onto the AOC platform for downstream applications.

### 2.8 Organoid growth boost

Human lung tissue is a limited source of cell material. For an increase in the material of AT2s, organoids grown from enriched AT2s were cultured in alveolar medium (alveolar), alveolar medium supplemented with 25% v/v EGM2 (+EGM2), or alveolar medium supplemented with 25% v/v conditioned EGM2 (+cEGM2). To condition the EGM2 for the production of cEGM2, HUVECS cultured in fresh EGM2 within a T-25 cell culture flask at 37 °C and 5% CO_2_. Medium exchange was done regularly every 2–3 days. Within the range of confluency (70% < confluency < 90%) and after two days of culture, the medium was harvested and filtered through a 0.22 μm filter using a syringe. Fresh EGM2 was filtered likewise, and each was added to supplement the alveolar medium. For eased image acquisition and later analysis, Matrigel™ domes of 8 μl were cultured in all three media conditions (alveolar, +EGM2 and +cEGM2) inside a 96-well plate with a glass bottom. The 200 μl of medium were exchanged every two days over a period of 14 days. All domes were imaged every 2 days after media exchange using an inverted Nikon microscope.

### 2.9 Confocal imaging of organoids

Organoid cultures in Matrigel domes were rinsed gently with PBS (Sigma-Aldrich) and then fixed using 4% paraformaldehyde (PFA, Invitrogen) for 1 hour at room temperature. Post-fixation, cultures underwent two gentle washes with PBS, ensuring minimal disturbance to the domes. Three additional gentle washes with PBS followed this step. Permeabilisation and blocking of the cultures were achieved using a solution of 0.05% Triton X-100 (Sigma-Aldrich) in 2% bovine serum albumin (BSA, Sigma-Aldrich) in PBS, with incubation for 2 hours. Subsequently, the cultures were subjected to three gentle PBS washes before incubation in the primary antibody staining solution for 24 hours. Following this, another set of three gentle PBS washes was performed, after which the cultures were incubated in the secondary antibody staining solution for another 24 hours. After a final set of three PBS washes, the prepared samples were imaged using a Nikon W1 LIPSI spinning disk microscope system.

### 2.10 Image Analysis and organoid size quantification using OrganoID

Organoids cultured as per previously described methods were subjected to bi-weekly imaging over a 14-day period. These images were processed using the OrganoID deep-learning platform, as detailed in a recent publication.^23^ Organoid segmentation was performed using the optimised model under default settings, and the images featuring labelled organoids were quantitatively analysed using CellProfiler software. The collected data underwent statistical evaluation using GraphPad Prism software (version 10). Statistical analysis involved the Kruskal-Wallis test for initial group differences in the data. Significant results were further analysed using the Mann-Whitney U test for pairwise comparisons. Significance levels were denoted as follows: ‘*’ for *p* < 0.05, ‘**’ for *p* < 0.01, ‘***’ for *p* < 0.001, and ‘****’ for *p* < 0.0001.

### 2.11 Flow-based weight measurement of organoids

In our study, we adopted a sophisticated method to evaluate the biophysical properties of organoids.^57,58^ It involved tracking the motion of organoids as they fell within a vertical flow channel with stationary fluid. This enabled us to measure their size and terminal velocity to determine the weight, size, and density of our organoids. The process involved the use of organoids, freed from Matrigel and 4% PFA fixed afterwards, which were resuspended in W8 solution (CellDynamics). The W8 system (CellDynamics) was initiated, and all tubes containing the organoids were installed according to the supplier's instructions. For the measurement of organoids cultured in alveolar medium, we defined the expected range of size, requested by the instrument, from 50–200 μm. Organoids grown in +EGM2 and +cEGM2 supplemented media we set the expected range of size to 100–400 μm. Following these measurements, we conducted a statistical comparison of the organoid weight, size, and density values using the analysis matrix supplied by CellDynamics. In our study, we adopted a sophisticated method to evaluate the biophysical properties of the cultured organoids. This approach allowed us to assess and compare the physical attributes of organoids under the three mentioned culture conditions.

### 2.12 On-chip cell culture and breathing experiments

AOC-chip plates were sterilised using an ozone-UV cleaner for 30 minutes prior to laboratory use. The AOCs were then pre-coated with polydopamine solved in Milli-Q water for 24 hours, followed by three washes in Milli-Q water. Subsequently, the AOCs were coated with 20 μl of a mixture of type 1 bovine collagen (50 μg ml^−1^, Advanced Biomatrix) and human fibronectin (100 μg ml^−1^, Corning) for 2 hours. This was followed by two washes with Milli-Q water and one wash with PBS. Alveolospheres were dissociated as outlined in the Culture of AT2 Organoids section. 25 000 AT2s were seeded per AOC in 70 μl of AM+E medium into the central chamber, with the addition of Y-27632 (10 μM, Sigma-Aldrich) for the first 24 h. The medium was replaced by DMEM (Sigma-Aldrich) containing 10% v/v FBS and 1 μg mL^−1^ Primocin (Invivogen) daily over a period of 6–7 days. Upon reaching confluency of the cell layer, the medium was changed, and the AOCs were pressurised from 0–400 mbar with sinusoidal breathing cycles 0.25 Hz for 24 hours. Total RNA was subsequently isolated using TRIreagent and prepared for RNA sequencing.

### 2.13 RNA sequencing and analysis

Total RNA was isolated from 6 samples using the Direct-zol RNA Microprep (Zymo Research). The quantity and quality of the purified total RNA was assessed using a Thermo Fisher Scientific Qubit 3.0 fluorometer with the Qubit RNA HS Assay Kit (Thermo Fisher Scientific, Q32855) and an Advanced Analytical Fragment Analyzer System using a Fragment Analyzer RNA Kit (Agilent, DNF-471), respectively. Sequencing libraries were made with 1 ng total RNA input using a Takara SMART-Seq mRNA Library Prep kit (Takara, 634769) in combination with Takara Unique Dual Indexes (Takara, 634752) according to the user manual from Takara (Takara, 100422). The cDNA and libraries were evaluated using a Thermo Fisher Scientific Qubit 3.0 fluorometer with the Qubit dsDNA HS Assay Kit (Thermo Fisher Scientific, Q32854) and an Agilent Fragment Analyzer (Agilent) with a HS NGS Fragment Kit (Agilent, DNF-474), respectively. The cDNA libraries were pooled and sequenced with a loading concentration of 300 pM, 100 bp paired-end and dual indexed, using a shared illumina NovaSeq 6000 1 lane of a S2 Reagent Kit v1.5 (200 cycles; illumina 20028315), using an XP workflow (illumina, 20043130) on an illumina NovaSeq 6000 instrument. The quality of the sequencing runs was assessed using illumina Sequencing Analysis Viewer (illumina version 2.4.7) and all base call files were demultiplexed and converted into FASTQ files using illumina bcl2fastq conversion software v2.20. The quality control assessments, the generation of libraries and sequencing was carried out at the Next Generation Sequencing Platform, University of Bern.

The RNA-seq data quality was assessed using fastqc v.0.11.9 (Andrews, bioinformatics.babraham.ac.uk/projects/fastqc/) and RSeQC v.4.0.0.^59^ Reads were mapped to the reference genome using the HiSat2 v.2.2.1 alignment tool.^60^ FeatureCounts v.2.0.1^61^ was used to count the number of reads overlapping with each gene as specified in the annotation of the genome annotation (Homo_sapiens.GRCh38.107). The Bioconductor package DESeq2 v1.38.3^62^ was used to test for differential gene expression between the experimental groups control and stretch. Gene set enrichment analysis (GSEA)^63^ was run in ClusterProfiler v4.7.1^64^ using gene sets from KEGG^65^ and MSigDb.^66^ All analyses were run in R version 4.2.1 (2022-06-23, r-project.org).

## 3. Results

### 3.1 Development of a novel alveoli-on-chip

**Fig. 1:**
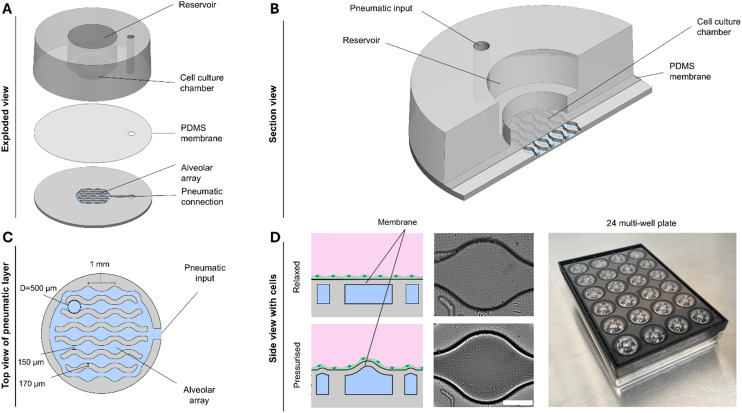
The novel alveoli-on-chip (AOC). A. Exploded view of the device showing the three main layers: the cell culture chamber with central reservoir, the thin PDMS membrane, and the pneumatically controlled alveolar array with external connection. B. Section view illustrating the apical cell culture chamber, the basal pneumatic input, and the flexible PDMS membrane separating the two compartments. C. Top view of the pneumatic layer showing the interconnected alveolar array with physiologically relevant dimensions (∼500 μm diameter alveoli, 150 μm interconnections). D. Left: Schematic side view showing the membrane with cells in the relaxed state (top) and under positive pressure forming hemispherical protrusions (bottom); center: Microscopy images confirming membrane deformation between relaxed and pressurized states; right: Photograph of the integrated 24-well plate format enabling parallelized experiments and compatibility with standard laboratory workflows.

During respiration, air flows in and out of the lungs due to the expansion and deflation of the thoracic cage, resulting in the stretching of the lung tissue. This mechanical force affects the cells of the lung, including those lining the alveolar epithelium.^18^ To mimic the alveolar strain, we developed a novel AOC incorporating an array of dimension-matched alveoli (Fig. 1).

The AOC device is microfabricated as a single piece of soft PDMS and consists of three layers: (i) a cell culture chamber and reservoir layer, (ii) a thin PDMS membrane layer, and (iii) a pneumatic layer (Fig. 1A). The apical culturing compartment and the basal pneumatic compartment are seamlessly separated by a 40 μm membrane that serves as the cell culture surface. The culture chamber includes a central reservoir for medium exchange, positioned directly above the flexible membrane.

Beneath this membrane lies the pneumatic compartment, which contains interconnected circular chambers with sinusoidal rims between adjacent cavities (Fig. 1B). This alveolar array is linked to an external pneumatic input that enables cyclic pressurization and relaxation. The array was designed with physiologically relevant dimensions, featuring alveoli of ∼500 μm in diameter with minimum interconnecting widths of 150 μm (Fig. 1C). This geometric configuration ensures that the resulting cyclic strain profile closely mimics that of native alveoli. When pressure is applied, the thin culturing membrane above each cavity expands into a hemispherical protrusion, resembling alveolar inflation. A positive deflection was intentionally chosen to prevent contact between the membrane and the compartment base, thereby allowing sufficient displacement while maintaining optimal imaging conditions through a short focal distance. Upon pressure release, the membrane recoils to its relaxed state (Fig. 1D, left). Live imaging confirmed the reversible membrane deformation and demonstrated that cultured epithelial cells were exposed to physiologically relevant three-dimensional strain (Fig. 1D, center).

For scalability and ease of use, the AOC system was integrated into a standard 24-well plate format (Fig. 1D, right), ensuring compatibility with conventional culture workflows and enabling higher throughput experimentation.

### 3.2 Fine-tuned pneumatic deflection

**Fig. 2:**
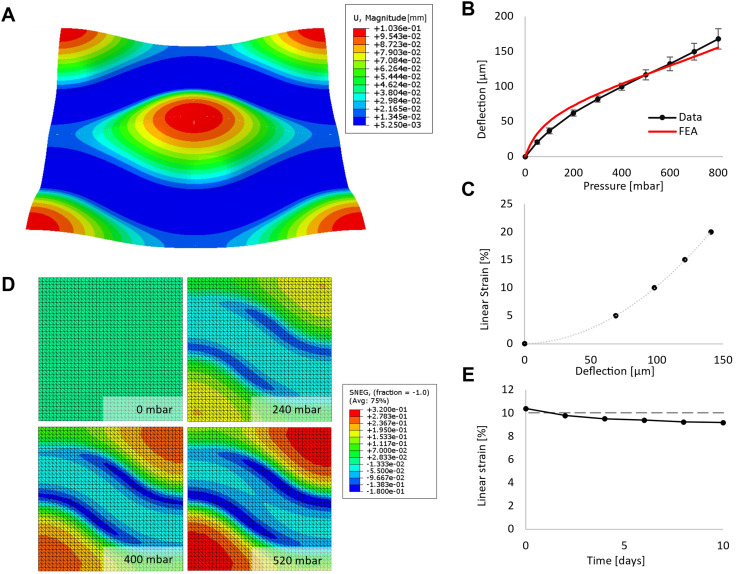
Mechanical characterisation of the novel AOC system. A. The deflected AOC's culturing surface, simulated by FEA. The colour code shows the amplitude of the deflected membrane. B. Pressure-deflection curve from 0 to 800 mbar shows the tuneable range of variable deflection. The plotted pressure-deflection curve of the FEA simulation (red) aligns closely with the measurements. C. Linear strain calculated from eqn S1 in function of the membrane deflection. D. Numerical simulation of the surface strain of the membrane in function of the pressure applied. Only a quarter of the culturing surface is simulated to exploit geometrical symmetry for greater simulation precision. E. Long-term deflection measurements. A cyclic pressure of 400 mbar peak was applied to the membrane 10 days at 37 °C, after a pre-conditioning time of 5 days at 37 °C.

To mimic respiratory cyclic stretch, the AOC is pressurised with a sinusoidal breathing pattern through the pneumatic inlet. The pressure is exerted towards the membrane separating the cell culture and pneumatic compartment, resulting in the deflection of the alveoli's culturing surface (Fig. 2A). The three-dimensional strain to which the cells are exposed is similar to the mechanical stress *in vivo* that occurs in the alveoli.^19^ However, to allow comparison with other currently used strain platforms that apply only one-dimensional strains,^2^ we can mathematically convert the surface strain by considering only the one-dimensional strain component (elongation).^20^ To characterise our device, the membrane was exposed to increasing pressure, and its deflection was measured microscopically (Fig. 2B and C). The strain experienced by the cells was defined using eqn S1. The simulated values obtained with the finite element analysis (FEA) model fit well with the experimental values for progressive pressure-driven deflection.^55^ According to this model, the maximal area strain is reached in the centre of an alveolus, while the minimum is reached at its rim on the channel wall (Fig. 2C and S2A). The deflection of the membrane ranges from 0 to 160 μm and can be precisely tuned by adjusting the air pressure. Specifically, at pressures of 0, 240, 400 and 520 mbar, the linear strain reaches 0, 5, 10 and 15%, respectively (Fig. 2B and D). To test the reproducibility of the membrane deflection over time, the membrane was pre-incubated for 5 days and then exposed to a sinusoidal pressure pattern of 400 mbar peak-to-peak and a frequency of 0.25 Hz for 10 days at 37 °C. The deflection of the membrane decreased slightly over time, which translates into a variation of the linear strain from 10.4 to 9.2% (Fig. 2E). For the 24-hour period of 10% cyclic stretch designated for later on-chip experiments within this study, the decline lies at 0.6% linear strain, from 10.4 to 9.8%. In addition, the incubation temperature of 37 °C did not affect the magnitude of deflection compared to measurements done the same day at room temperature (Fig. S2B).

### 3.3 AT2 organoid culture for the expansion of patient-derived cells

To set the stage for our findings, Fig. S1 provides an overview of the experimental workflow. The process begins with the isolation of primary AT2 cells, which were expanded as patient-derived organoids (alveolospheres). These organoids were then dissociated and seeded onto the alveolus-on-chip platform, where cyclic stretch was applied to mimic physiological conditions, followed by transcriptomic analyses to capture cellular responses. This framework guided all subsequent experiments presented in the following.

**Fig. 3:**
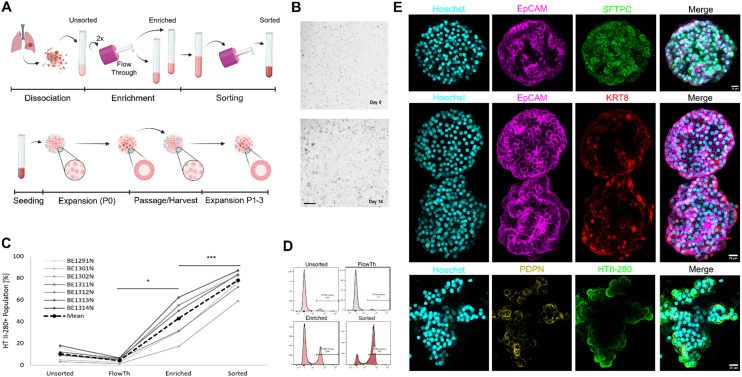
Alveolar epithelial cell organoid culture and characterization. A and B. Human lung tissue resections are dissociated, cells isolated, enriched and sorted, followed by cell expansion *via* organoids and passaging for harvest or further expansion. Scale bar for B: 500 μm. C and D. FACS is used to assess the enrichment and sorting percentage (positive/total) of the isolated AT2 populations. The numbers BE1291N to BE1314N correspond to the individual patients from whom the cells were sorted and cultured for expansion in organoids. E. Maximum intensity projection (MIP) images of organoids stained for epithelial cells (EpCAM, magenta), AT2s (SFTPC, HTII-280, green), AT1s (PDPN, yellow), and AT0s (KRT8, red). Nuclei are counterstained with Hoechst (cyan). Organoids display a predominantly epithelial architecture containing AT2s, AT1s, and AT0s subtypes. Scale bars: 20 μm.

AT2 were sorted from human lung cancer resections that were histologically classified as not involved in the tumour. These tissues were dissociated, and the AT2s magnetically sorted and seeded in Matrigel to expand their populations in the form of alveolospheres (Fig. 3A and B). We enriched the AT2 population through a two-step magnetic sorting procedure. First, the unsorted cell suspension was subjected to two rounds of enrichment, followed by a final sorting step of the combined enriched fractions (*cf.* STAR methods). The use of a test tube-based sorting magnet instead of a column magnet improved ease of use and handling time. On average, the sorting protocol yielded 74.03% HTII-280^+^ cells (AT2) from the unsorted population (Fig. 3C, D and S3A). Donor-to-donor variability in the initial AT2 fraction was observed, as expected for primary human samples, but this had no significant effect on organoid formation, and the number of isolated AT2s was sufficient to initiate organoid formation. After several passages, organoids were immunostained with markers for epithelial cells (EpCAM), AT1s (PDPN), AT2s (SFTPC, HTII-280), and AT0s (KRT8) to determine their composition. The results are shown in Fig. 3E as maximum intensity projections, and in Fig. S3B as a single optical section revealing the characteristic hollow alveolosphere. These analyses demonstrated that the organoids were composed predominantly of epithelial cells, including AT2s as well as AT1s and AT0s subtypes.

### 3.4 Conditioned medium to boost alveolar organoids growth

**Fig. 4:**
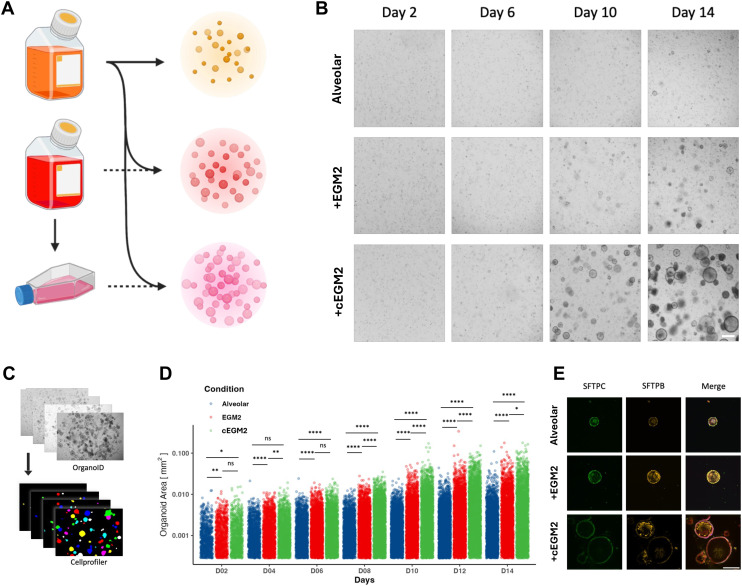
AT2 organoids growth boosted A. Schematic of media composition strategy with alveolar medium (100% alveolar) supplemented with fresh (25% EGM2) or conditioned (25% cEGM2) endothelial growth medium. B. Microscopical images of AT2 organoids cultured in pure or supplemented media, show distinct differences in size. Scale bar: 500 μm C. brightfield images are segmented and analysed using the OrganoID^23^ segmentation platform and a Cellprofiler software. D. Quantification of the organoid cross-sectional area for all mentioned conditions (alveolar, EGM2 and cEGM2 supplemented). Each dot represents one measured organoid. The organoids were grown over a 14-days period and are presented as a dot plot with a logarithmic *y*-axis. *N* = 5 patients, with *n* = 3 to 7 replicates for each. The significance intervals are denoted as ns for not significant, * for *p* < 0.05, ** for *p* < 0.01, *** for *p* < 0.001, and **** for *p* < 0.0001. E. Confocal images of AT2 organoids grown for 14 days in the mentioned conditions (alveolar, EGM2 and cEGM2 supplemented). Stained for SFTPC, SFTPB, actin, and nuclei. Scale bar: 200 μm.

AT2 organoid cultures take 4 to 6 weeks to grow and reach a viable size and population for cell harvesting.^21^ This long period of time impedes the frequency of experimentation and thus is a bottleneck for work involving human material. To overcome this limitation, we followed the idea of a supplement-based growth boost, previously described by Ravi *et al.* 2022.^22^ For this purpose, AT2 organoid cultures received either the established formulation of the alveolar medium without further supplements (alveolar), alveolar medium supplemented with fresh endothelial growth medium (+EGM2) or alveolar medium supplemented with conditioned endothelial growth medium (+cEGM2) (Fig. 4A). To generate the conditioned medium, endothelial cells (HUVEC cell line) were cultured for two days, after which their medium supernatant was harvested (*cf.* STAR methods). Compared to the alveolar medium, the AT2 organoid cultures grew faster with both supplemented media (Fig. 4B). Overall, the largest organoids were observed in the conditioned media supplement (cEGM2). Further analysis based on image segmentation using the deep-learning platform OrganoID^23^ enabled subsequent quantification (Fig. 4C). The size of the organoids differed significantly between the conditions (Table S1). The organoids grown in alveolar medium displayed the smallest average sizes of 2791.67 μm^2^. The organoids supplemented with EGM2 and cEGM2 showed significantly larger sizes, with an average of 5478.75 μm^2^ and 7425.81 μm^2^, respectively (Fig. 4D). This resulted in a 1.96 and 2.66-fold increase in organoid size for EGM2 and cEGM2 supplements, respectively, compared to the size of organoids at day 14 cultured without these supplements. We further measured the organoid density and size using a physical cytometer (W8 system) (Fig. S4). A significant difference is observed between organoids cultured in the alveolar medium compared to the organoids grown with supplemented media. However, no significant difference was observed between the conditions +EGM2 and +cEGM2. In all conditions, the organoids consistently express the AT2 markers of SFTPC and SFTPB (Fig. 4E).

### 3.5 The impact of cyclic stretch on the alveolar epithelium

**Fig. 5:**
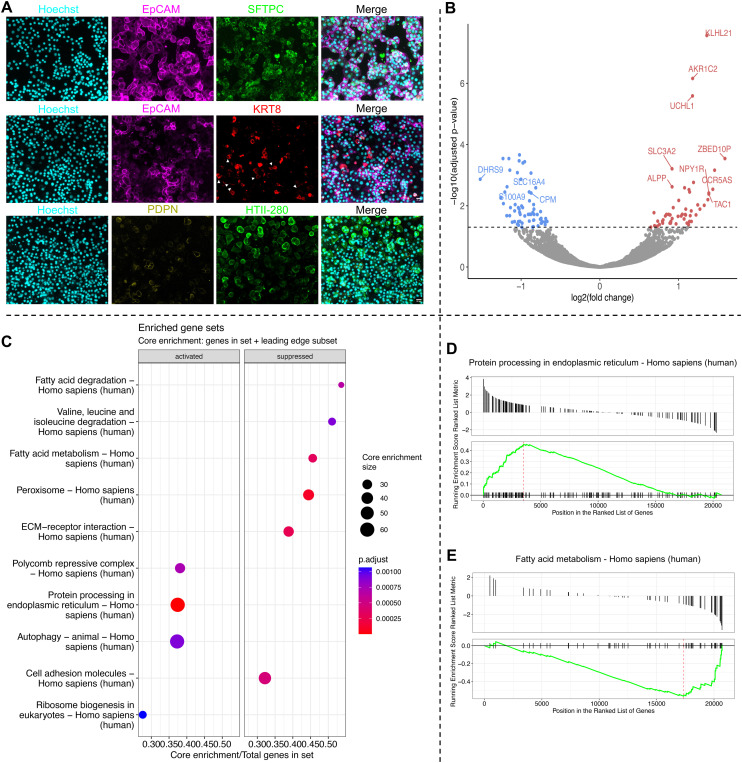
Transcriptomic and immunostaining analysis of alveolar epithelial cells cultured on the AOC. A. After 6 days of culture on PDMS membranes, organoid-derived cells established an epithelial layer. Immunostaining confirmed the presence of multiple alveolar epithelial subtypes, including AT2 (SFTPC^+^, HTII-280^+^), AT1 (PDPN^+^), and transitional AT0 (KRT8^+^), with EpCAM (magenta) marking epithelial identity and Hoechst (cyan) counterstaining nuclei. Scale bars: 20 μm. B. Volcano plot overview of the differentially expressed genes. Some of the genes with the highest adjusted *p*-value and log2 fold-change are annotated. C. Gene set enrichment analysis (GSEA) overview dot-plot showing the activity or suppression of significant KEGG gene sets. D and E. GSEA showing the activity or suppression of stretch-affected gene sets.

After the expansion phase, organoids were harvested and dissociated into single-cell suspensions. These cells were subsequently seeded on the PDMS membrane of the AOC chip, where they formed an epithelial layer within 6–7 days, consisting of a mixture of AT2, AT1, and transitional AT0 subtypes (Fig. 5A). A physiological cyclic respiratory stretch was then applied for 24 hours. After 24 hours of breathing stimulation, RNA was isolated from stretched and non-stretched samples and analysed *via* RNA sequencing. The application of cyclic stretch induced significant changes in gene expression. Among the differentially expressed genes, we observed downregulation of carboxypeptidase M (CPM), a known marker of lung epithelial progenitors and AT2 cells,^24^ consistent with mechanically induced cellular maturation responses described in the literature.^25^ Genes such as Kelch like family member 21 (KLHL21), involved in ubiquitination and cytokinesis,^26^ Aldo–Keto reductase family 1 member C2 (AKR1C2), the ubiquitin C-terminal hydrolase L1 (UCHL1), a protease that targets C-terminal glycine of ubiquitin^27^ and nebulin (NEB), a stabiliser of F-actin were upregulated (Fig. 5B and S5C). Concurrently, genes encoding for a metalloreductase (STEAP4),^28^ ganglioside GM3 synthase (ST3GAL5),^29^ and the transcriptional and immune response regulator (TCIM)^30^ presented downregulation under mechanical stretch.

In addition to these gene-specific alterations, our analysis identified several cellular processes affected by respiratory stretch, shown by a gene set enrichment analysis (GSEA) (Fig. 5C, E and S5D–H). In detail, processes such as protein processing within the endoplasmic reticulum, activities of the polycomb repressive complex (PRC), and autophagy were positively influenced by dynamic stretching (Fig. 5C and D). Conversely, pathways involved in the degradation and metabolism of fatty acids, the degradation of amino acids (valin, leucin, isoleucin), and cell adhesion molecules were downregulated (Fig. 5C and E). Further examination of the transcriptional changes of pathways described within the molecular signature database revealed stretch-associated upregulation of the G2M checkpoint, mTORC1 signalling, and the unfolded protein response (Fig. S5C and E). In contrast, pathways such as the interferon-alpha response, coagulation, and epithelial–mesenchymal transition showed downregulation under mechanical stretch (Fig. S5D and E).

## 4. Discussion

The lung/alveolus-on-chip technology has already offered great insights into pulmonary physiology and promising improvements to preclinical models.^9,11,20,31^ These systems are aimed at replicating one or several features of the alveolar microenvironment, such as the mechanical stress of the breathing motions, the air-liquid interface, or the interactions with other cells.^32,33^ These developments led, among others, to a deeper understanding of how mechanical cues influence cell fate and the physiology of the alveolar epithelium.^8,16,34^

Our aim was to develop a robust alveoli-on-chip (AOC) system able to tuneable reproduce an array of tiny alveoli exposed to 3D breathing movements to investigate the effect of the cyclic mechanical stress on the lung alveolar epithelium at both the cellular and transcriptomics levels. The new system incorporates an array of alveoli with a diameter of 250 μm, which results in an alveolar surface area close to the alveoli *in vivo*^35,36^ and reproduces a more complex stress gradient profile observed *in vivo*.^37^ The reduction in alveolar diameter, as opposed to previously reported systems with a 3 mm diameter membrane,^38^ results in a decreased membrane deflection when keeping the same linear strain. This reduction enables a shorter focal length and, thus, the direct imaging of cells cultured on the membrane without having to disassemble the system. This simplifies the handling of the AOC considerably, as it can be easily imaged microscopically. Moreover, the open-top design is another feature that simplifies the handling of cell culture, and its microplate format makes it compatible with standard laboratory equipment such as microscopes. It should also be emphasised that the chip design facilitates an increased throughput to 24 wells compared to other lung-on-chip models.^38,39^ However, it's worth noting that some of these models have additional properties, such as the ability to create an air-liquid interface, which our system lacks. Consequently, neither trans-epithelial electrical resistance (TEER) measurements nor permeability assays can be performed due to the use of a non-porous membrane. However, the system can be modified to incorporate a self-organised microvasculature of endothelial cells and lung fibroblasts embedded in a thick hydrogel layer on the chip that can be cyclically stretched (see Fig. S6).^40^ This modification would allow lung alveolar epithelial cells to be cultured on the hydrogel layer in which the microvasculature is formed, so that an air–blood barrier could be created without the need for a membrane between the endothelial cells and the alveolar epithelium, while still permitting mechanical stretching.^41^ The increased throughput of the current AOC is particularly noteworthy as it addresses the challenge of achieving higher throughput in organ-on-chip systems.^42^

The AOC's performance under varying pressure conditions and over extended periods of time demonstrates the system's robustness and reliability. The close alignment of the simulated and measured values for progressive pressure-driven deflection further validates the AOC's ability to replicate alveolar strain.

Having introduced the AOC system's unique capabilities in replicating the cyclic mechanical stress to which the alveolar epithelium is exposed, we turned our attention to establishing a relevant source of primary human alveolar epithelial cells to ensure that we could generate enough cells for our research. We modified an isolation protocol for the organoid generation aimed at increasing the AT2 population.^21^ Instead of isolating AT2 cells by FACS or column-based magnetic-activated cell sorting (MACS) methods,^16,43^ we used a tube-based MACS approach. These adaptations resulted in a sufficient percentage of AT2 cells to generate organoids, while also accelerating the enrichment process. The resulting alveolar epithelial organoids contained a mixture of AT1, AT2, and AT0 populations. However, the expansion period required for these alveolospheres ranged from 4 to 6 weeks, depending upon the patient material. While this time frame proved sufficient to obtain enough cells for harvesting, it is a slow and inefficient process counterproductive for experiments we wanted to improve. Inspired by the work of Ravi *et al.*^22^ who were able to increase the alveolar organoids by 2.3-fold within 10 days by culturing endothelial cells at the bottom of inserts in which primary AT2 cells were cultured to generate AT2 organoids. We tested the efficiency of an endothelial-conditioned medium for faster growth of alveolospheres. Cellular crosstalk in the form of biomolecular signals is known to be crucial for the proliferation and maintenance of AT2s. Previously, a number of studies highlighted the impact of epithelial–mesenchymal crosstalk.^44–46^ Other works focused on the contribution of the endothelium to the alveolar epithelial niche.^47,48^ Our approach of using only the endothelial supernatant is based on the hypothesis that endothelial supplied alveolar potentiators (ESAP) are predominantly spread *via* paracrine or endocrine signalling and thus might increase the efficiency of AT2 expansion. This approach would allow the exclusion of endothelial cells from alveolar culture and thus reduce the risk and complexity of co-culture approaches. Therefore, we cultured alveolospheres within the feeder-free media conditions: alveolar medium and the alveolar medium supplemented with EGM2 or cEGM2. Both supplemented conditions showed an increase in cell yield compared to the alveolar medium, with 1.96-fold and 2.66-fold increases for EGM2 and cEGM2, respectively. The 2.5-fold increase observed in cEGM2 medium after 10 days of culture is similar to that obtained by Ravi and colleagues.^22^ This increase in yield is likely caused by mitogenic factors within the supplemented media.

The dissociated cells from the organoids were cultured on-chip, resulting in a mixed population of AT1s, AT2s, and AT0s after 7 days (Fig. 5A). Our investigations reveal that cyclic respiratory stretch significantly influences gene expression in the human alveolar epithelium. We observed an increase in the expression of genes associated with autophagy, unfolded protein response, G2M checkpoint, and mTORC1 signalling, indicating a generalised cellular stress response. The autophagy-related regulation of inflammatory mediators within the alveolar epithelium has been observed previously^49^ as well as the involvement of respiratory stretch.^50^ Thus, our findings of increased autophagic activity with a decreased expression of inflammatory response genes (Fig. S5 G and H) are in line with previous findings. While the concurrent upregulation of mTOR activity and autophagy may seem unusual, a previous study described the biaxial stretch-related mTOR-independent induction of autophagy in a cell model of eye cells.^51^ In line with these mechanobiological effects, we also observed downregulation of carboxypeptidase M (CPM), a marker of lung epithelial progenitors and AT2 cells.^24^ This decrease likely reflects mechanically induced differentiation of alveolar epithelial cells from progenitor states toward more mature phenotypes, rather than pathological changes, consistent with the broader pattern of homeostatic responses observed in our study.^3,25^

Additionally, our data suggests the involvement of the polycomb repressive complex PRC, indicating an epigenetic aspect in cellular adaptation to mechanical stretch. This is supported by the work of Quang Le and colleagues, who highlighted the mechanical control of transcription and polycomb-mediated gene silencing.^52^ Furthermore, our study presents the downregulation of genes involved in epithelial–mesenchymal transition, inflammation, cell metabolism, differentiation, and signalling. This suggests that respiratory stretch plays an active role in maintaining the homeostasis of the alveolar epithelium.^53^

Beyond advancing our fundamental understanding of alveolar mechanobiology, this integrated platform of patient-derived alveolospheres and organ-on-chip technology offers promising potential for personalized medicine applications.^54^ The ability to culture patient-specific alveolar epithelial cells under physiologically relevant mechanical conditions could enable personalized drug screening, disease modeling, and therapeutic optimization tailored to individual patient responses.

## 5. Conclusion

In this study, we introduced a novel alveoli-on-chip model capable of fine-tuned application of respiratory stretch over extended periods. We additionally developed an optimised method for culturing human alveolospheres, enabling faster expansion of AT2 cells and facilitating more efficient cell harvest—a key advancement for research applications. Utilising the innovative system in combination with organoid-expanded human AT2 cells, we investigated the impact of cyclical stretch on the alveolar epithelium and unveiled new insights into the transcriptional changes associated with recreating physiological cellular microenvironments.

## Author contributions

O. T. G., J. S. and M. A. H. designed the research; J. S. designed the novel alveoli-on-chip device; J. S., D. P. and D. S. fabricated the novel alveoli-on-chip devices; J. S. prepared, cultured, and applied stretch to the alveoli-on-chips; D. P., D. S., T. W. and J. S. designed the numerical simulation of the alveoli-on-chip; D. P. and D. S. conducted the numerical simulation of the alveoli-on-chip; P. D. conducted the surgeries to retrieve donor materials; T. M. M. tested, delivered and managed the donor materials; J. S., S. S. and M. A. H. isolated, J. S. established, and J. S. and M. A. H. maintained and imaged the alveolospheres; J. S. initialised, optimised, and imaged the alveolosphere growth boost; J. S., J. H. L and O. T. G. designed the AT2 on-chip experiments; J. S., O. T. G., M. A. H. and P. Z. performed data analysis; J. S., M. A. H., O. T. G wrote the manuscript; O. T. G. commented and critically revised the manuscript; all authors reviewed the manuscript.

## Conflicts of interest

J. S. and O. T. G. are listed as inventors on a patent application pertaining to the alveoli-on-chip design described in this study. All other authors declare no competing interests.

## Supplementary Material

LC-025-D5LC00473J-s001

## Data Availability

Hajari *et al.* a novel alveoli-on-chip platform for modeling cyclic stretch in patient derived alveolar epithelial cells cultured from organoids, submitted to Lab Chip. The RNA Seq data are available at Zenodo: DOI: https://doi.org/10.5281/zenodo.10953365. The data originates from lung tumor samples obtained at the University Hospital Bern. Patient-derived alveolar epithelial type 2 cells (AT2) were isolated and cultured into organoids. Grown organoids were dissociated and seeded onto a novel alveoli-on-chip system submerged in medium. After 24 h of either no mechanical stimulus (control) or cyclic breathing stretch (stretch), RNA was isolated and sequenced. The RNA-seq data underwent quality control, alignment to the reference genome, read counting, DGE and GSEA. All analyses were run in R version 4.2.1. The CAD files of the AOC chip are available at Zenodo: DOI: https://doi.org/10.5281/zenodo.17224123. Supplementary information (SI) is available. See DOI: https://doi.org/10.1039/d5lc00473j.
