# Supplementary material for: A novel alveoli-on-chip platform for modeling cyclic stretch in patient-derived alveolar epithelial cells cultured from organoids
Source: Lab Chip. 2025 Oct 24;25(24):6688–702. doi: 10.1039/d5lc00473j (PMC12593192; doi:10.1039/d5lc00473j)
Supplement: LC-025-D5LC00473J-s001 [file LC-025-D5LC00473J-s001.pdf]

Supplementary Information (SI) for Lab on a Chip.
This journal is © The Royal Society of Chemistry 2025

A Novel Alveoli-on-Chip Platform for Modeling Cyclic Stretch in Patient-Derived Alveolar Epithelial Cells Cultured from Organoids

Mohammad Amin Hajari[1,2], Jan Schulte[1,2], Dario Principi[1], Damian Schnidrig[1], Sabine Schneider[1], Tobias Weber[1,2], Joo Hyeon-Lee[3,4], Patrick Dorn[5,6], Pauline Zamprogno[1], Thomas Michael Marti[5,6], Olivier T. Guenat[1,6,7]

1 Organs-on-Chip Technologies Lab, ARTORG Center, University of Bern, Bern, Switzerland
2 Graduate School for Cellular and Biomedical Sciences (GCB), University of Bern, Bern, Switzerland
3 Cambridge Stem Cell Institute, Jeffrey Cheah Biomedical Centre, University of Cambridge, Cambridge, CB2 0AW, UK
4 Developmental Biology Program, Sloan Kettering Institute, Memorial Sloan Kettering Cancer Center, New York, NY 10065, USA
5 Department of BioMedical Research, University of Bern, Bern, Switzerland
6 Department of General Thoracic Surgery, Inselspital, University Hospital of Bern, Bern, Switzerland
7 Department of Pulmonary Medicine, Inselspital, University Hospital of Bern, Bern, Switzerland

Supplementary Information

Equation S1:

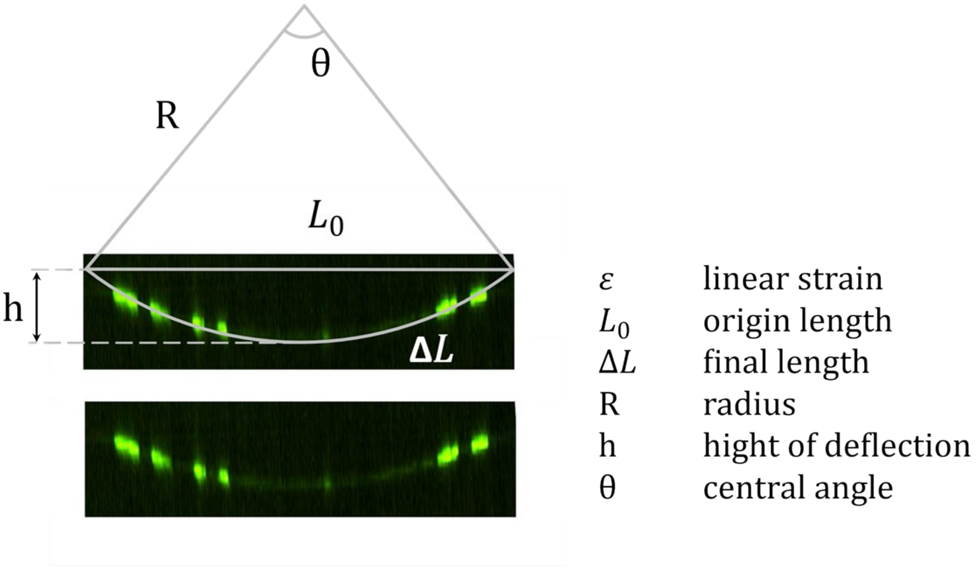

Equation S1a: Linear strain

$$\varepsilon = \frac{\Delta L}{L_0}$$

Equation S1b: Radius of a circular segment:

$$R = \frac{h}{2} + \frac{{L_0}^2}{8h}$$

Equation S1c: Central angle of a circular segment

$$\theta = 2\arcsin\frac{L_0}{2R}$$

Equation S1d: Arc length of a circular segment

$$L_0 = \theta R$$

Equation S1e: Arc length from a, b, c and d

$$L_0 = \frac{\left({L_0}^2 + 4h^2\right) \cdot arcsin\left(\frac{4L_0h}{{L_0}^2 + 4h^2}\right)}{4h}$$

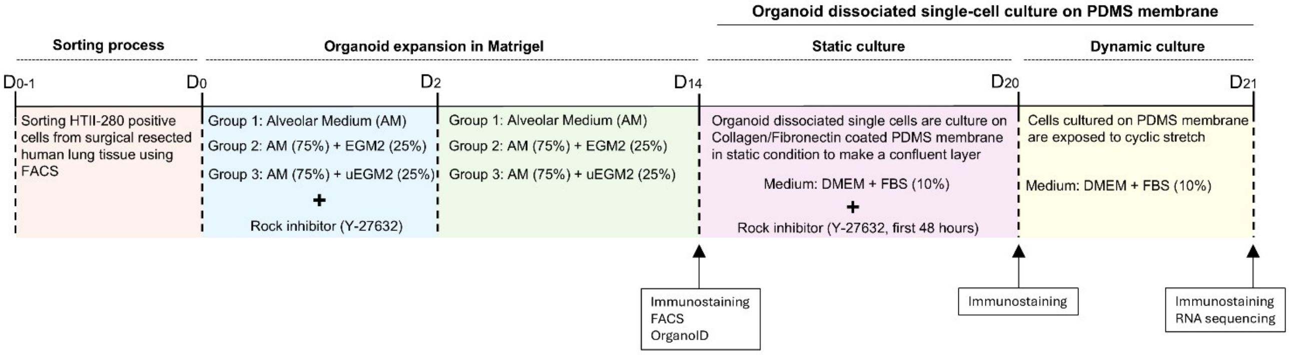

Figure S1 Schematic representation of the experimental workflow, including isolation of HTII-280–positive cells from human lung tissue, organoid expansion in Matrigel, dissociation and seeding of single cells onto PDMS membranes, and subsequent static or dynamic culture conditions.

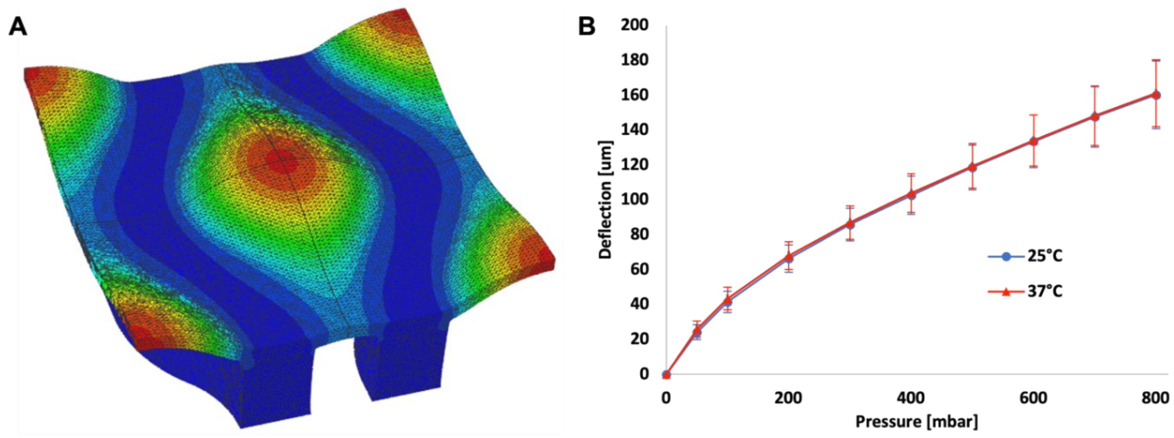

Figure S2 Characterisation of fine-tuned membrane stretch. **A** The meshed simulation model used to generate the finite element analysis. **B** Pressure-deflection graph illustrating that the incubation temperature does not affect the deflection compared to measurements done at room temperature.

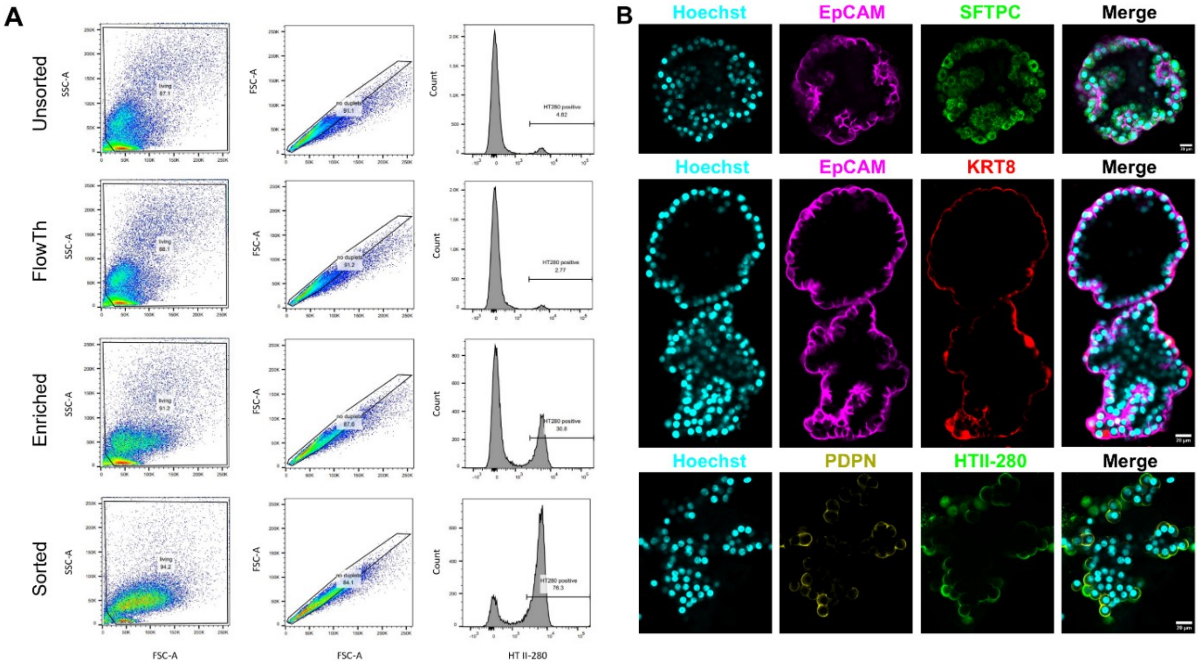

Figure S3 A Detailed FACS plots of an enrichment-sorting experiment. **B** Representative optical section of organoids stained for epithelial cells (EpCAM, magenta), AT2 (SFTPC, HTII-280, green), AT1 (PDPN, yellow), and AT0 (KRT8, red). Nuclei are counterstained with Hoechst (cyan). In contrast to the maximum intensity projections shown in Fig. 3E, this section illustrates the spatial distribution of distinct epithelial subtypes within the hollow structure of the alveolosphere. Scale bars: 20 µm.

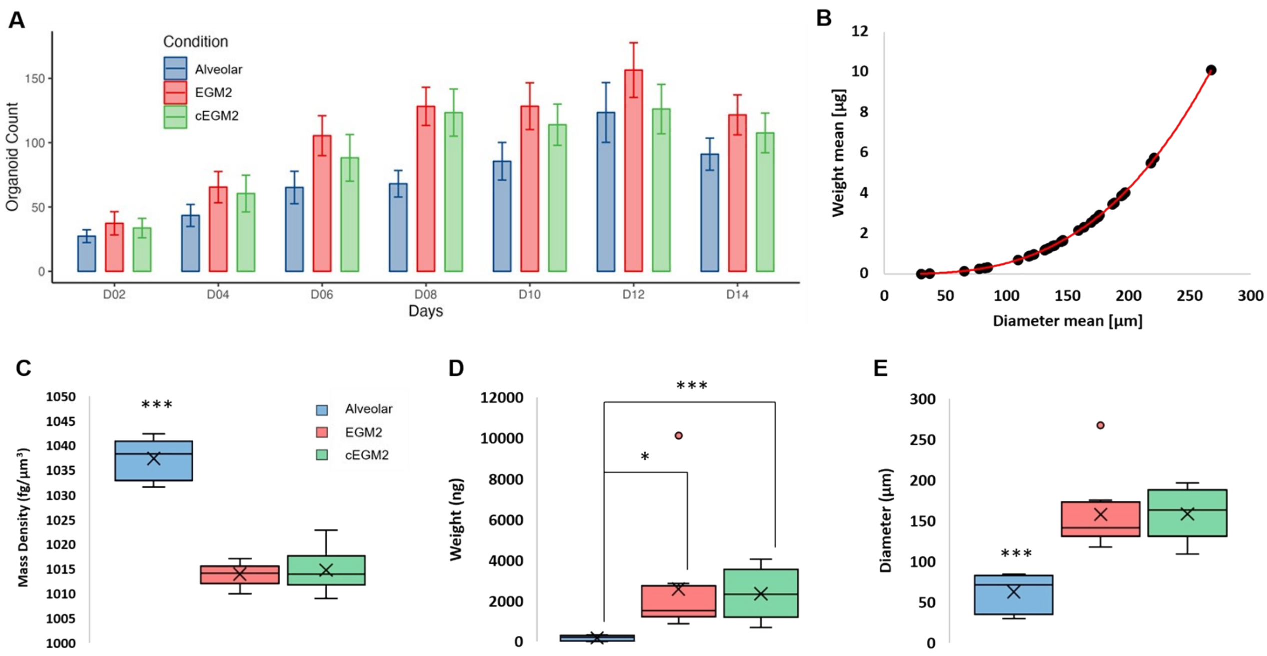

Figure S4 AT2 organoid growth boost analysis alternative approach. **A** Quantification of the organoid number for all mentioned conditions (Alveolar, EGM2 and cEGM2 supplemented) using OrganoID. Each bar represents the mean number of organoids from the imaged hydrogel domes of one condition on the respective day of culture. The organoids were grown over a 14-day period. Error bars represent the standard error of the mean. **B** Diameter-weight dot-plot of 14-day-old Organoids combined from all conditions. The red line represents a polynomial 2nd order trendline with $y = 0.0006x^{2.9814}$ and $R^2 = 1$. **C-E** Measurements of alveolospheres from all three conditions (Alveolar, EGM2 and cEGM2 supplemented). Diameter and weight are direct measurements, while the device calculates mass density from these values. Measurements from B-E were done with W8 CellDynamics Cytometer

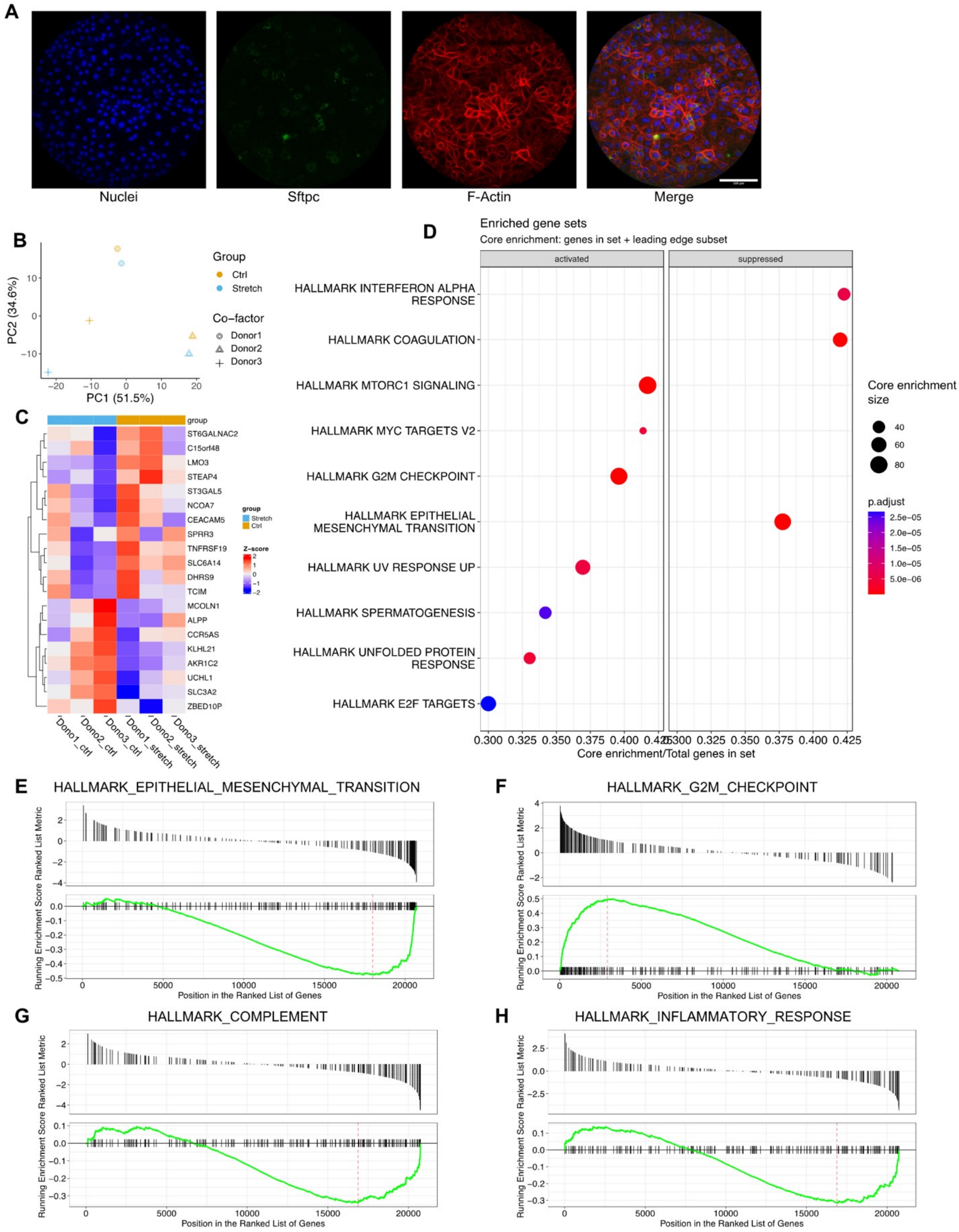

Figure S5 Supplementary transcriptomic profiling of the cyclically stretched alveolar epithelium. **A** Confocal immunostaining image of alveolar epithelial cells seeded on the novel AOC and cultured for 7 days. Nuclei (blue), SFTPC (green) and F-Actin (red) are detected. **B** Principal component analysis plot of the first two axes based on the 500 most variable genes. **C** Group-wise heatmap of the most differentially expressed genes based on the highest

adjusted p-value and log2 fold-change are annotated. **D** Gene set enrichment analysis (GSEA) overview dot-plot showing the activity or suppression of significant gene sets from the molecular signature data base. **E**-**H** GSEA showing the activity or suppression of stretch-affected gene sets from the molecular signature data base. Epithelial-mesenchymal transition (D), G2M-checkpoint (E), complement cascade (F) and inflammatory response (G).

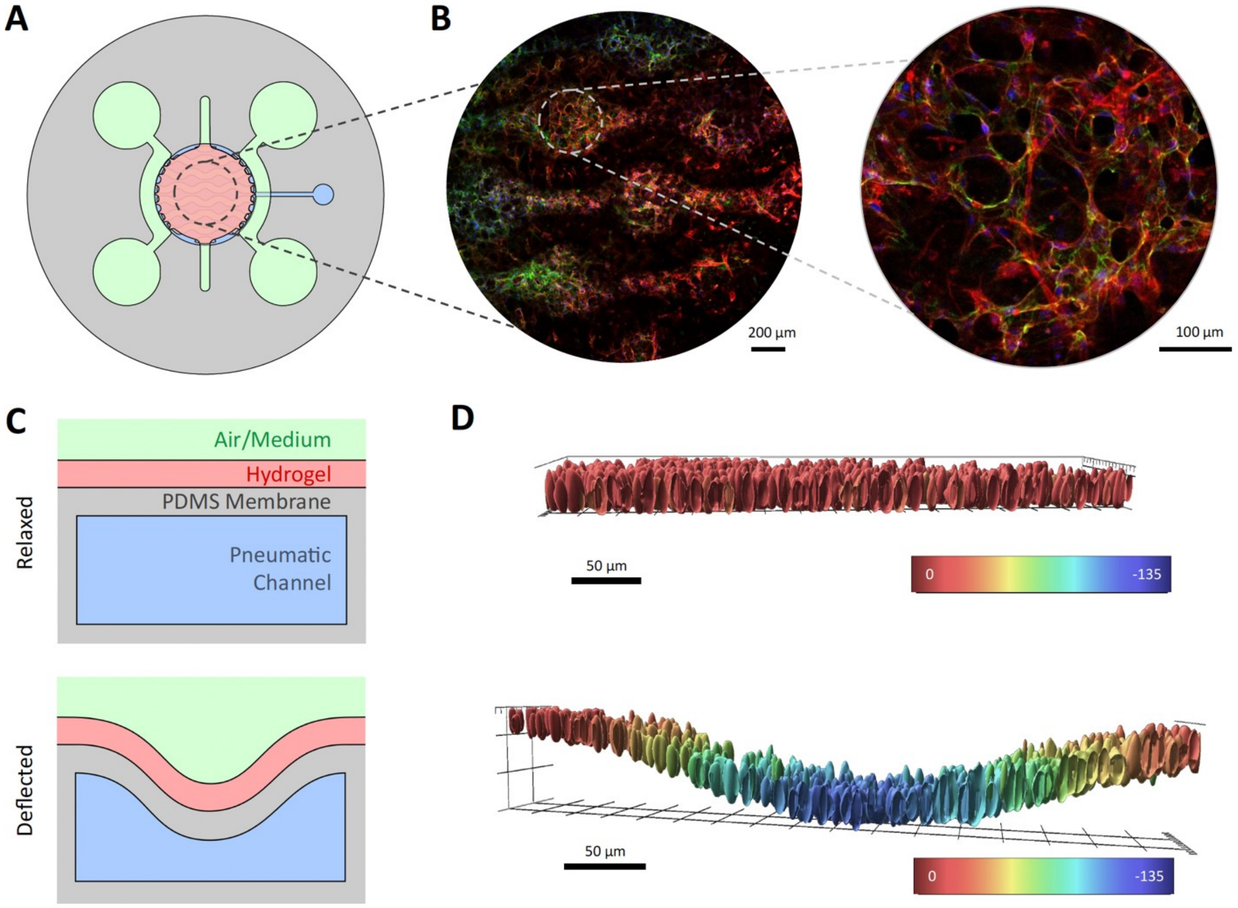

Figure S6 A Chip with a hydrogel layer, **B** incorporating a self-assembled microvasculature made of endothelial cells (HUVEC) and human lung fibroblasts after 5 days. **C** Schematic cross-section of the chip at rest and deflected. **D** Cross-section of the deflected gel (rendering based on confocal imaging and Imaris).

Table S1 AEC organoid growth boost area measurement means.

[μm^2]	Alveolar	EGM2	cEGM2
D02	1158.685	1381.024	2266.538
D04	1355.656	1670.072	1608.26
D06	1576.369	1899.406	2037.299
D08	1712.969	2367.019	3170.084
D10	2044.362	3435.806	5108.158
D12	2457.711	4629.968	6930.819
D14	2791.67	5478.747	7425.811
fold-change at day 14 compared to Alveolar:		1.963	2.66

KEY REAGENT/RESOURCE TABLE

REAGENT or RESOURCE	Source	Identifier
Epoxy plastic metal Weicon C	Weicon	10100005
PDMS, SYLGARD 184	Dow Corning	1317318
PBS	Sigma-Aldrich	D1283
4% PFA	Invitrogen	I28800
BSA	Sigma-Aldrich	A9418
EGM2 Bulletkit	Lonza	CC-3162
Dispase II	Sigma-Aldrich	D4693
Collagenase type 4	Worthington	LS004189
DNase I	Sigma-Aldrich	D4527
FBS	Sigma-Aldrich	F7524
Penicillin-Streptomycin	Invitrogen	15140122
Red Blood Cell Lysis buffer	BioLegend	420301
Advanced DMEM/F-12	Thermo Fischer Scientific	12634010
HEPES	Sigma-Aldrich	H3375
Primocin	Invivogen	ant-pm-1
N-Acetylcysteine	Sigma-Aldrich	A9165
Nicotinamide	Sigma-Aldrich	N0636
Matrigel GFR PhRed-free LDEV-free	Corning	356231
B27 serum-free supplement	Invitrogen	0080085SA
rhFGF-10	Peprotech	100-26
rhEGF	Peprotech	AF-100-15
rh KGF (FGF-7)	Peprotech	100-19
rhNOGGIN	Peprotech	120-10C
SB431542	Sigma-Aldrich	616461
CHIR99021	Sigma-Aldrich	SML1046
Y-27632 dihydrochloride	Sigma-Aldrich	Y0503
DMEM	Sigma-Aldrich	D6171
TrypLE	Thermo Fischer Scientific	12604039
Type I bovine Collagen	Advanced Biomatrix	5005
Human fibronectin	Corning	356008
TRI Reagent	ZYMO RESEARCH	R2061
Direct-zol RNA Microprep	ZYMO RESEARCH	R2061

Antibodies

REAGENT or RESOURCE	Source	Identifier
Anti-HT II-280 mouse IgM	Terrace Biotech	TB-27AHT2-280
Anti-mouse IgM MicroBeads	Miltenyi Biotec	130-047-302
Anti-mouse IgM AF488	Invitrogen	A-21042
Anti-SPC conj. AF488	Santa Cruz Biotechnologies	sc-518029 AF488
Anti-Hop conj. AF546	Santa Cruz Biotechnologies	sc-398703 AF546
Anti-RAGE/AGER goat IgG	R&D systems	AF1145
Anti-proSPB rabbit	Seven Hills	WRAB-48604
Hoechst 33342	Sigma-Aldrich	14533
Acti-stain 670 phalloidin	Cytoskeleton	PHDN1-A
Anti-CD326 (EpCAM) conj. efluor 660	Invitrogen	50-9326-41
Anti-PDPN conj. AF647	SantaCruz	sc376695
Anti-Cytokeratin 8	Sigma-Aldrich	MABT329M

CONTACT FOR REAGENT AND RESOURCE SHARING

Further information and requests for resources should be directed to and will be fulfilled by the lead contact, Prof. Dr. Olivier T. Guenat (olivier.guenat@unibe.ch)
